# Maternal Effects May Act as an Adaptation Mechanism for Copepods Facing pH and Temperature Changes

**DOI:** 10.1371/journal.pone.0048538

**Published:** 2012-10-31

**Authors:** Anu Vehmaa, Andreas Brutemark, Jonna Engström-Öst

**Affiliations:** 1 ARONIA Coastal Zone Research Team, Novia University of Applied Sciences and Åbo Akademi University, Ekenäs, Finland; 2 Tvärminne Zoological Station, Hanko, Finland; University of Hamburg, Germany

## Abstract

Acidification of the seas, caused by increased dissolution of CO_2_ into surface water, and global warming challenge the adaptation mechanisms of marine organisms. In boreal coastal environments, temperature and pH vary greatly seasonally, but sometimes also rapidly within hours due to upwelling events. We studied if copepod zooplankton living in a fluctuating environment are tolerant to climate change effects predicted for 2100, i.e., a temperature increase of 3°C and a pH decrease of 0.4. Egg production of the copepod *Acartia* sp. was followed over five consecutive days at four temperature and pH conditions (17°C/ambient pH; 17°C/low pH; 20°C/ambient pH; 20°C/low pH). Egg production was higher in treatments with warmer temperature but the increase was smaller when copepods were simultaneously exposed to warmer temperature and lowered pH. To reveal if maternal effects are important in terms of adaptation to a changing environment, we conducted an egg transplantation experiment, where the produced eggs were moved to a different environment and egg hatching was monitored for three days. When pH changed between the egg production and hatching conditions, it resulted in lower hatching success, but the effect was diminished over the course of the experiment possibly due to improved maternal provisioning. Warmer egg production temperature induced a positive maternal effect and increased the egg hatching rate. Warmer hatching temperature resulted also in earlier hatching. However, the temperature effects appear to be dependent on the ambient sea temperature. Our preliminary results indicate that maternal effects are an important mechanism in the face of environmental change.

## Introduction

Increased atmospheric CO_2_ concentration is causing global warming. As CO_2_ dissolves in surface water, carbonate chemistry changes and the concentration of dissolved inorganic carbon increases, thereby decreasing pH and causing ocean acidification [1]. Coastal organisms are subjected to a large array of environmental stressors during their lifetime. In upwelling regions, both surface water temperature and pH can drop substantially within hours when old deep-water is replaced and forced to the surface. Species that inhabit such fluctuating environments are likely to be tolerant to ocean acidification [2]. Further, preceding acidification events or regular pH changes can cause strong selection for evolution of acid tolerance [3].

Maternal effects can be important as adaptations to environmental stress. Maternal effect are defined as cross-generation phenotypic plasticity, implying the capability of a mother to adjust the phenotype of her offspring as response to environmental cues that her offspring will encounter, in a manner that enhances offspring fitness [4]–[5]. One of the most studied maternal effects of this type is offspring size (Bryozoan: [6], Ural owl: [7], Sydney rock oyster: [8]). When environmental conditions are non-optimal, it is better to produce a few larger, high quality and fast developing offspring, whereas the number of offspring should be maximized in extremely favorable conditions because all perform well, regardless of size [4], [6]. For example, Sydney rock oyster larvae are larger and develop faster in higher CO_2_ conditions, if the adults also have been incubated in high CO_2_ conditions [8].

In laboratory studies, calanoid copepods have indicated high tolerance to increased CO_2_ concentrations [9]–[10]. A seawater pH decrease of 0.6–0.9 did not substantially affect survival of *Acartia* copepods [9], [11]. The responses seem to vary between life stages; a pH decrease between 1.0 and 1.3, which did not affect the survival rates of adult copepods, impacted negatively on egg viability and larval development [11]–[12]. However, a pH decrease of 0.41 was enough to reduce juvenile production of a harpacticoid copepod in an experiment using three generations [13]. Ocean acidification acts in concert with other climate change factors such as temperature, UV-radiation, and salinity [14]. In our previous study, we combined lowered pH (−0.4 pH) with two temperatures (17°C and 20°C) in accordance with a 2100-scenario [1], [14]. We found that copepod female antioxidant capacity decreased at the warmer temperature and lowered pH (Vehmaa et al. unpublished manuscript). In addition, maternal oxidative damage was negatively related to production of viable eggs, and maternal antioxidant capacity had a positive effect on juvenile development, but not on egg production rate. Rodríguez-Graña et al. (2010) found a similar maternal effect that was related to female age; older females produced fewer eggs with lower hatching success, and the resulting offspring had higher protein oxidative damage [15].

Here we further tested the reproductive response of *Acartia* sp. calanoid copepods and the importance of maternal effects in determining the offspring quality in a changing environment according to a 2100 climate scenario of a pH decline by 0.4 units and a temperature elevation of 3°C [1], [13]. We hypothesized that copepods in the Gulf of Finland (Baltic Sea) are tolerant to such temperature and pH changes because even, the seasonal variability in their environment can be much larger [16]–[17], and that this tolerance, which is seen also in other parts of the world [9]–[10], is due to the ability of the copepod to invest in its eggs and adjust them accordingly for best performance for prevailing conditions. To test this, we monitored egg production of copepods incubated in four different pH and temperature conditions for five consecutive days. On days 1, 3 and 5, we conducted an egg transplantation experiment, divided the produced eggs and allowed them to hatch in either the same or in different conditions than those in which they were produced. We expected a higher hatching success when the eggs were hatching in the same environment as that in which they were produced.

## Methods

### Test Organisms

The study took place in mid-August 2011. Zooplankton were collected using a 200 µm plankton-net equipped with a 1 l cod-end by vertical net hauls from 25 m depth at Storfjärden monitoring station (Hanko, Finland). The monitoring station is located on a nature reserve owned by the University of Helsinki, and permission for research was granted by Tvärminne Zoological Station (University of Helsinki). The collected animals were immediately transferred to 30 l containers with approximately 20 l of water and transported to the laboratory. Healthy looking *Acartia* sp. copepod females and males were immediately thereafter sorted randomly into 1.2-l Duran clear glass bottles, containing treatment water. We assumed all the copepods to be *A*. *bifilosa*. However, since *A*. *tonsa* was also present at the sampling site we cannot totally exclude the possibility of having a few of them in the treatments. Sorting was completed within 8 h after copepod sampling.

During the experiment copepods were fed with the cryptophyte *Rhodomonas* sp. (strain 07B6), which was maintained as non-axenic batch cultures in f/2 medium without silica [18] at 17°C in 8.2 µmol photons m^−2^ s^−1^ with 16∶8 h light:dark regime. In addition, the copepods were fed with Bioplankton (Liquid Life, Gardena, CA 90248, USA), a commercially available and nutritious food solution, consisting of *Isochrysis* sp., *Nannochloropsis* sp. and *Tetraselmis* sp. Bioplankton was kept in the freezer (−12°C) until used. The diets were prepared by mixing *Rhodomonas* sp. (268.2±2.5 µg C l^−1^, 51.8±0.7 µg N l^−1^; mean ± SD) and Bioplankton (198.1±9.0 µg C l^−1^, 21.6±0.2 µg N l^−1^), corresponding to ∼470 µg C l^−1^, in total. Samples of *Rhodomonas* sp. and Bioplankton cells were filtered onto pre-combusted (450°C, 4 h) GF/F Whatman filters and their particulate organic carbon (POC) and nitrogen (PON) were analyzed with a mass spectrometer (Europa Scientific TracerMass, upgraded with ANCA 20-20 parts).

### Experimental Design

Copepods were exposed to four different environments: 1) 17°C and ambient pH, 2) 17°C and lowered pH, 3) 20°C and ambient pH, and 4) 20°C and lowered pH (Table 1). Seawater was collected at the same site as the animals (surface 19°C, pH 8.17 and salinity 5.8 ppt) and prepared by sterile filtering (<0.2 µm, Sartobran 300 filters; Sartorius Stedim Biotech GmbH. Göttingen, Germany). The experiment was performed using two temperature controlled rooms: 17°C as control (August upper mixed layer average [19]) and 20°C (+3°C from the 2100 scenarios [14]). After reaching the target temperature, water for treatments with lowered pH was supplemented with CO_2_ until pH decreased ∼0.4 units. Dissolved oxygen was measured from the treatment waters with YSI Environmental Pro ODO™ meter. Saturation point was always >90%. Light intensity, measured at the top of the bottles with a LI-COR LI-1000 light meter, was ∼9 µmol photons m^−2^ s^−1^ with 16∶8 h light:dark regime in both rooms.

**Table 1:** Water pH (min – max (median)) of the replicate bottles at the start and at the end of the egg production incubation, and the replicate Petri dishes at the end of the egg hatching incubation.

	Egg production			Egg hatching	
	Start	End	n	End	n
17°C ambientpH	7.65–8.27 (8.13)	7.51–8.11 (7.76)	45	7.32–7.88 (7.51)	72
17°C low pH	7.31–7.60 (7.49)	7.26–7.55 (7.39)	15	7.30–7.50 (7.40)	24
20°C ambientpH	8.02–8.30 (8.11)	7.97–8.14 (8.08)	15	7.48–8.04 (7.87)	24
20°C low pH	7.50–7.67 (7.58)	7.49–7.69 (7.60)	15	7.52–7.88 (7.72)	24

The control treatment was 17°C with ambient pH and had 9 replicates; the other treatments were triplicated. Twenty females and three males were incubated in a 1.2-l Duran clear glass bottle, which was completely airtight. The copepods were collected every ∼24 h onto a 250 µm sieve and live copepods were transferred back to the same bottle with a renewed treatment water and food suspension. pH was measured (TUNZE, pH-controller 7070/2) in each bottle prior to closing and immediately after opening (Table 1). Eggs were collected from each bottle by filtering them onto a 38 µm mesh-size sieve. The collected eggs were reared in 50 ml Petri dishes, filled with treatment water and sealed without airspace using Parafilm.

**Figure 1:**
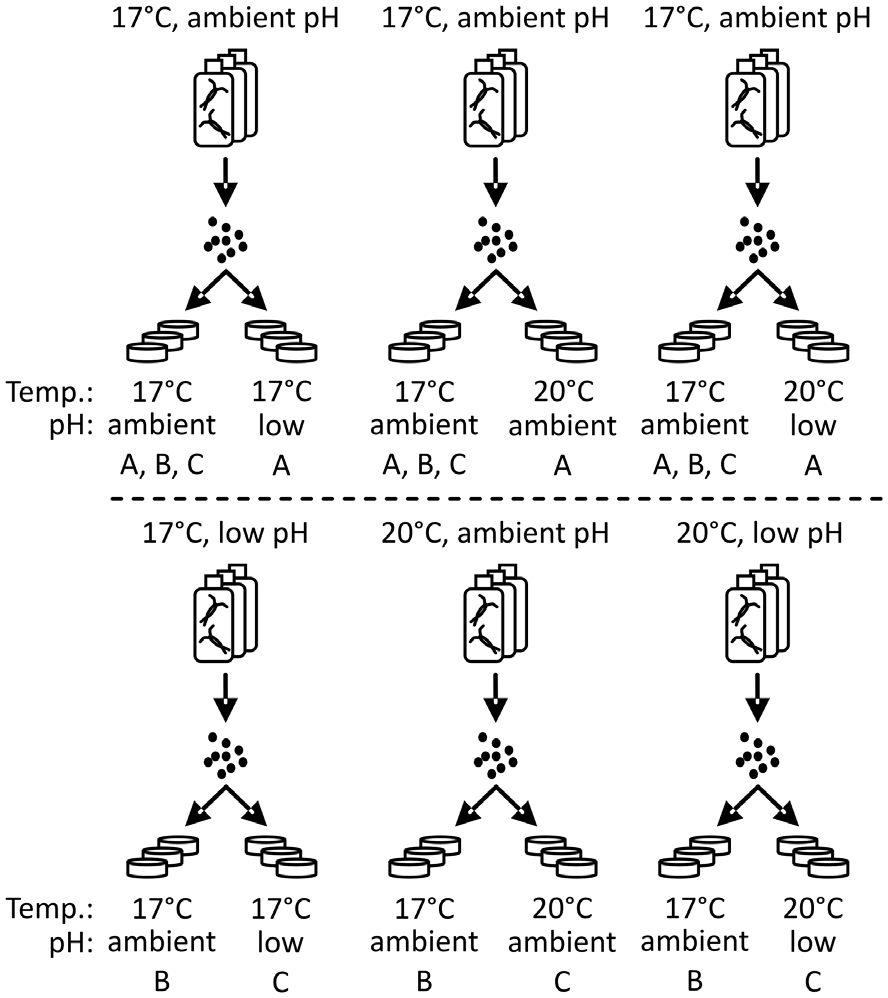
Set-up of egg production and egg-transplantation experiment. After egg production on days 1, 3 and 5 the eggs were divided for hatching in two different conditions. The number of bottles and Petri dishes in the figure are equivalent to the experiment. Eggs are A) produced at 17°C ambient pH on days 1, 3 and 5, but hatched in different conditions, B) produced in different conditions on days 1, 3 and 5, but hatched at 17°C ambient pH, C) hatched in same conditions that they are produced on days 1, 3 and 5.

In the egg-transplantation experiment on days 1, 3 and 5, collected eggs were split in half and one half was incubated in the egg production environment and the other half was transferred to a different environment (Fig. 1). Egg hatching was monitored twice daily during a ∼72 h period by using dissecting microscopes to count the remaining eggs on the Petri dish. Eggs collected on days 2 and 4 were reared in the same conditions as they were produced in (eggs were not split). After a ∼72 h hatching period, acid Lugol’s solution was added, the hatched copepod juveniles (hereafter nauplii) and unhatched eggs were counted. Egg production rate (EPR), expressed as number of hatched and unhatched eggs produced per live female during 24 h, and nauplii production rate (NPR) were calculated. Hatching success was calculated as percentage of number of nauplii divided by the sum of nauplii and unhatched eggs found in the samples after addition of Lugol. pH was measured from each Petri dish at the end of hatching (Table 1). Copepods that survived the five-day egg production experiment (81%) were released to the sea. No other permits than the permit to work in a nature reserve were required for the described studies, and the studies did not involve endangered or protected species.

### Statistical Analyses

The combined egg (∑^5^_i = 1_EPRi) and nauplii production (∑^5^_i = 1_NPRi) data were analyzed using two-way factorial ANOVA with the factors temperature, pH (two levels) and their interaction. Because it varied between days but less within-treatment than between treatment, pH was treated as a factor in the analysis. Model assumptions, i.e., constancy of variance and normality of errors were checked after fitting the model by using the Fligner-Killeen test, and by plotting the residuals against fitted values, and standardized residuals against theoretical quantiles. All statistical analyses were conducted using software R 2.10.1 [20].

Egg production during five consecutive days was analyzed using a linear mixed effects model (LMM) with restricted maximum likelihood (REML) approximation using the nlme-package [21]. Temperature (two-level factor), pH (arithmetic averages for start and end H^+^ concentrations converted back to the pH scale), day and all their two-way interactions were used as fixed effects. Because the interaction between temperature and pH was significant, indicating that the effect of pH is different in the two temperatures, the model was rerun to establish estimates of pH effect at 17°C and at 20°C [22]. The random effect structure was day (repeated measure) within each bottle. Model simplification was done manually in a backward stepwise manner using Akaike’s information criterion (AIC) and likelihood ratio test for justifying the simplifications. We report F-statistics of the retained fixed effects. After fitting the best possible LMM, residual diagnostics were performed to check that the assumptions were not violated.

The cumulative hatching of eggs produced on days 1, 3 and 5 was analyzed using a generalized linear mixed effects model (GLMM) with Laplace likelihood approximation using the lme4-package [23], with a binomial error structure and a logit link function [24]. The variables used in the full model, and their definitions are listed in Table 2. Model simplification was done manually in a backward stepwise manner using AIC and χ^2^-test. Interactions between the factors egg production temperature and hatching temperature and the covariate hatching time, and the factor day and the covariate |ΔpH| were significant, indicating that the relation between the covariate and hatching success differs between groups, and that a difference between groups depends on the value of the covariate [22]. The interactions prevent interpretation of the main effects of covariates. Since the only covariate of interest is |ΔpH|, we ran the model again for all three factor levels of day without the interaction, and without the day nested within bottles’ random structure.

**Table 2:** Variables that were used in the full hatching success model, and their definitions. * = interaction term.

Variable type	Variable	Definition
Fixed effects	Production pH, Production Temperature^2^,Prod pH * Prod Temp^2^	Does production environment affect egg hatching?
	Production pH * Time, Production Temp^2^ * Time	Does production environment affect egg hatching rate?
	Production pH *Day^3^ Production Temp^2^ * Day^3^	Does the effect of production environment change over time (acclimatization)?
	Hatching pH, Hatching Temperature^2^, Hatch pH * Hatch Temp^2^	Does hatching environment affect egg hatching?
	Hatch pH * Time, Hatch Temp^2^ * Time	Does hatching environment affect egg hatching rate?
	|ΔpH|, |Δtemp|^2^	Do differences in pH and temperature conditions between egg production and egg hatching environment affect hatching?
	|ΔpH|*Day^3^, |Δtemp|^2^*Day^3^	Does the effect of pH and temperature change between egg production and egg hatching environment differ between days?
Random effects	Day nested within levels of bottles(1|Bottle^18^/Day^3^)	Eggs that come from same bottle are more alike than other eggs, and eggs that come from same bottle on same day (divided into 2 Petri dishes) are even more alike.
	Repeated measures of same eggs counted over hatching time (Time|Petri_dish^108^)	Eggs on a Petri dish are counted several times, and thus are not independent replicates.

1, 2, 3, 18 & 108 = The number of factor levels.

## Results

**Figure 2:**
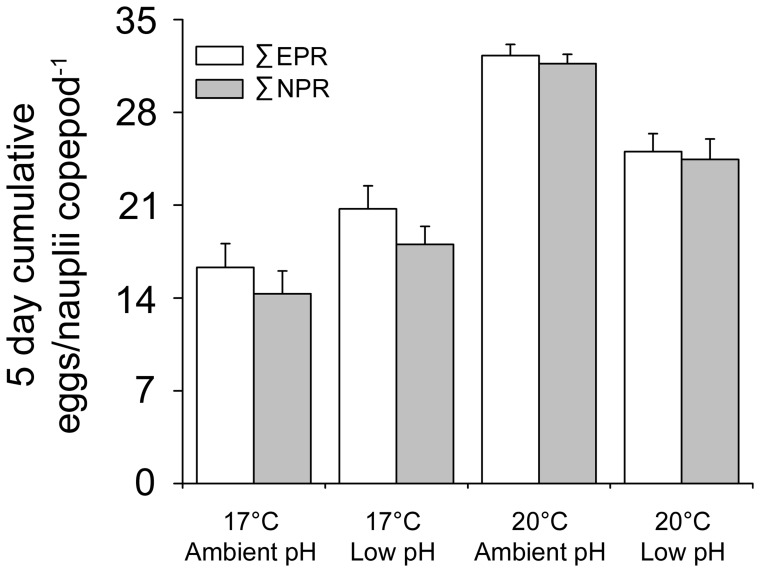
The combined egg (∑^5^_i = 1_EPRi) and nauplii production (∑^5^_i = 1_NPRi) in different treatments during the course of the study. Eggs were produced and hatching in the same conditions. N = 9 for ambient pH 17°C treatment and N = 3 for the other treatments. Values are given as mean ± SE.

The interaction between temperature and pH affected the total egg production during the five days (∑^5^_i = 1_EPRi ) significantly (Table 3); lower pH increases egg production at 17°C, but decreases egg production at 20°C (Fig. 2). Total egg production increased significantly in warmer temperature (Fig. 2), whereas pH alone did not affect egg production (Table 3). For total nauplii production (∑^5^_i = 1_NPRi ), the effect of temperature and pH interaction corresponded to the total egg production (Fig. 2). Warmer temperature also resulted in significantly higher number of nauplii, whereas pH had no significant effect (Table 4).

**Table 3:** ANOVA table for testing the sum of eggs produced in the course of the study.

	DF	SS	MS	F	P
Temperature	1	506.83	506.83	26.84	<0.001***
pH	1	0.25	0.25	0.013	0.910
Temp * pH	1	122.40	122.40	6.482	0.023*
Residuals	14	264.37	18.88		

**Figure 3:**
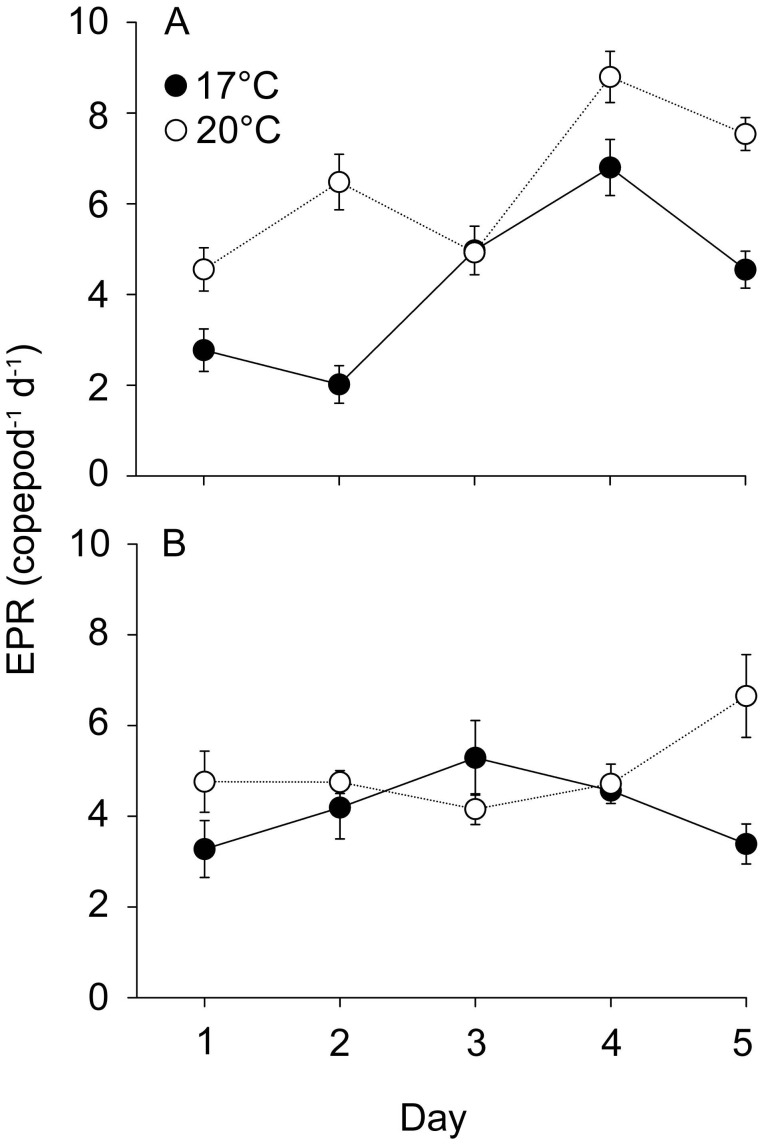
Egg production rate on five consecutive days in four different pH and temperature conditions. a) ambient pH and b) low pH with temperature 17°C (closed symbols) or 20°C (open symbols). N = 9 for 17°C ambient pH treatment and N = 3 for the other treatments. Values are given as mean of eggs copepod^−1^ day^−1^± SE.

Copepod egg production increased during the experiment in all treatments (LMM: *F*_1,69_ = 7.103, *P* = 0.010; Fig. 3). pH as a single factor did not affect egg production (LMM: *F*_1.69_ = 0.269, *P* = 0.606), but temperature had a significant effect (LMM: *F*_1,16_ = 27.266, *P*<0.001). The interaction between temperature and pH was also significant (LMM: *F*_1,69_ = 8.258, *P* = 0.005), indicating that the effect of pH on egg production differs between the two temperatures, and therefore, egg production is different at the two temperatures (Fig. 3). A more detailed look at the pH effect in the two temperatures reveals that egg production might increase with decreasing pH at 17°C (estimate 0.78±0.81 SE), whereas egg production decreases with pH at 20°C (estimate −2.90±0.78 SE).

**Figure 4:**
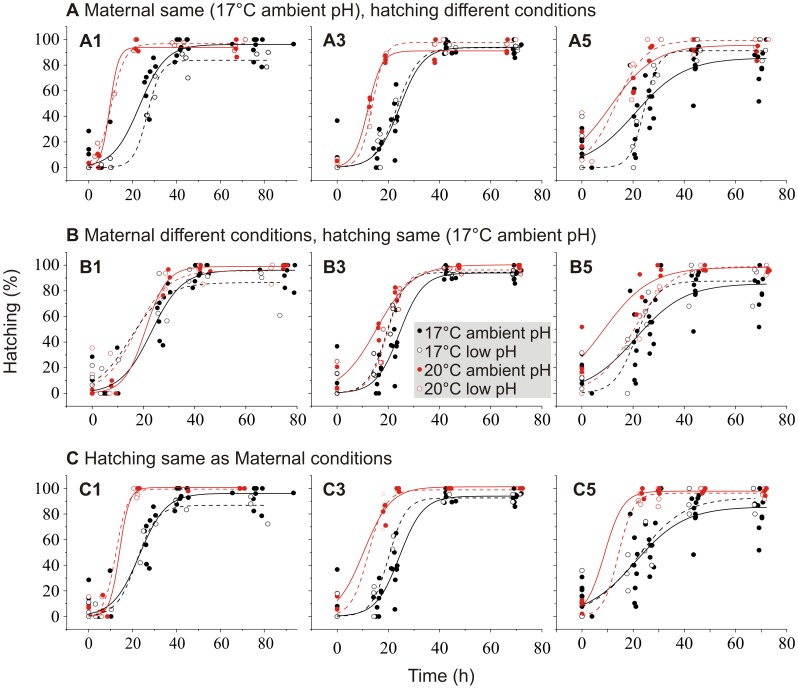
Cumulative hatching of eggs in transplantation experiment. Eggs are A1, A3 A5) produced at 17°C ambient pH on days 1, 3 and 5, but hatched in different conditions, B1, B3, B5) produced in different conditions on days 1, 3 and 5, but hatched at 17°C ambient pH, C1, C3, C5) hatched in same conditions that they are produced on days 1, 3 and 5. Best fit, sigmoidal curves: black line = 17°C ambient pH, black dashed line = 17°C low pH, red line = 20°C ambient pH, red dashed line = 20°C low pH.

Eggs hatched sooner when they were produced at 20°C compared with 17°C (Table 5: Prod temp*Time; Fig. 4: red curves compared to black curves). Also, warmer hatching temperature increased egg hatching rate (Table 5: Hatch temp*Time). The effect of hatching temperature was slightly larger than the effect of egg production temperature (compare estimates in Table 5). The significant interaction between the absolute difference in pH and day (|ΔpH|*Day) indicates that hatching success differed between the days 1, 3 and 5. The separate analyses for the days revealed that the difference in pH between egg production and hatching conditions resulted in lower hatching success on the first day (GLMM: *Z* = −2.615, *P* = 0.009; Fig. 4a and 4d), whereas it resulted in a higher hatching success on the third day (GLMM: *Z* = 2.279, *P* = 0.023) (Fig. 4b and 4e). The effect of pH difference on hatching had disappeared by the fifth day (GLMM: *Z* = 1.409, *P* = 0.159; Fig. 4c and 4f).

**Table 4:** ANOVA table for testing the sum of nauplii produced in the course of the study.

	DF	SS	MS	F	P
Temperature	1	656.51	656.51	38.11	<0.001***
pH	1	1.62	1.62	0.094	0.764
Temp * pH	1	107.79	107.79	6.257	0.025*
Residuals	14	241.19	241.19		

**Table 5:** The best statistical model for egg hatching success. Egg hatching was analysed using GLMM for binomial (hatched, unhatched) data including days 1, 3 and 5.

	Estimate	SE	Z-value	P-value
Intercept	−2.319	0.262	−8.82	<0.001***
Production Temperature (low)^ME^	0.027	0.239	1.13	0.259
Time	0.209	0.011	18.63	<0.001***
Hatching Temperature (low)	−0.068	0.233	−0.29	0.771
|ΔpH|^ME^	−0.942	0.443	−2.13	0.033*
Day 3	−0.382	0.214	−1.78	0.075
Day 5	−0.316	0.247	−1.28	0.200
Prod Temp (low)*Time^ME^	−0.051	0.011	−4.54	<0.001***
Hatch Temp (low)*Time	−0.067	0.012	−5.80	<0.001***
|ΔpH|*Day 3^ME^	1.897	0.710	2.67	0.008**
|ΔpH|*Day 5^ME^	1.698	0.630	2.70	0.007**

ME = The single and interaction variables indicating maternal effects.

## Discussion

Our preliminary estimates suggest that copepod reproductive output is sensitive to changes in both temperature and pH; total egg production was higher in treatments with warmer temperature, but the increase was smaller when copepods were simultaneously exposed to warmer temperature and lowered pH. Also, the egg production temperature affected hatching so that the eggs developed faster when they were produced at warmer temperature. The copepod females can thus possibly provision their eggs better in warmer water. Further, our results indicate that the eggs produced in the beginning of the study were of poorer quality than the eggs produced on day 5. Therefore, the environment change (|ΔpH|) was more detrimental for them. We consider the effect of the pH change (|ΔpH|) to be kind of a pre-zygotic maternal effect [5].

Negative effects of lowered pH on copepod reproduction have been found only at considerably lower pH than in the present study [11]–[12]. The egg production from five consecutive days together with the combined egg and nauplii production data, give clear indications that copepods incur costs from the interaction effect of temperature and pH. As life expectancy of adult copepods is short, only between days and weeks depending on temperature [25], the results from our five-day experiment suggest that these costs could lead to decreased lifetime reproduction success and reduced population sizes. Although the warmer temperature seemed to benefit copepod reproduction, we could speculate that the interaction with lowered pH might have narrowed the thermal window [26] and led to lower egg production. Temperature range that supports the optimal copepod growth can be gauged by examining egg production since energy of adult copepods is allocated to metabolic costs and to reproduction, not to somatic growth [27]. In addition to reducing upper thermal tolerance limits, increased CO_2_ concentration and temperature interaction decrease crustacean larval survival, increase adult mortality and decrease haemolymph oxygen concentration [2].

As a short-term study using one generation of copepods, these results do not take into consideration possible longer term adaptation potential. As a species with a short generation time, *Acartia* might be able to respond to the environmental changes through adaptive evolution [28]. Also, maternal effects might themselves be heritable. Copepod daughters might inherit their mother’s ability to provision their eggs [29].

Both warmer egg production temperature and hatching temperature increased egg hatching rates throughout the experiment. It is beneficial for eggs of broadcast-spawning copepods to hatch fast. If a sinking *A*. *bifilosa* egg encounters anoxic conditions it will become quiescent and postpone hatching until the conditions are favorable again [30]. However, the risks of being buried in anoxic sediments for good [31], or to be consumed by predators in water or sediments [32], are reduced as development rate increases. Further, early hatchlings that continue fast development during the nauplii stages gain advantage in food competition when resources are limited. Although slightly above their optimal temperature [33], 20°C is close to ambient surface water temperature in late summer [16]. A pilot study in June, when the water was colder, showed indications of an opposing temperature effect; egg production was lower at 20°C than at 17°C (data not shown). The length of our experiment was long enough to enable observation of acclimatization to the experimental conditions. However, the natural conditions prior to the experiment set limits on the acclimation potential and affect the results because copepod body size is temperature-dependent [34].

Adjusting offspring quality in a plastic manner can be adaptive for a mother in a fluctuating environment, but such a response is possible only if the expected offspring environment can be evaluated by the mother [35]. The egg transplantation experiment showed that a difference in pH between egg production and hatching conditions decreases hatching, which suggests that the eggs might be adjusted to certain environmental conditions. Water pH changes used in this study are irrelevant for copepod egg hatching when environmental cues are stable, as they are when eggs are produced and hatched in the same conditions.

Adult copepods and their offspring can experience rapid changes in both temperature and pH due to diel vertical migration, water mixing or upwelling events. Despite this, the temperature difference between production and hatching conditions did not negatively affect the reproductive output. Instead, higher production temperature induced a positive maternal effect resulting in faster hatching and indicating that the mothers can invest more in their eggs, and therefore produce better quality eggs. The different response to pH and temperature might arise from different thermal and acid-base regulation mechanisms.

The effect of pH difference between egg production and hatching environment on hatching success faded during the study when the copepods were acclimatizing to the experiment conditions. Feeding conditions were optimal enabling the adults to invest substantially in their offspring. However, in natural conditions food resources are fluctuating and there might not always be energy reserves available to allocate for cross-generational effects [8]. Therefore, copepods might be more vulnerable for pH changes during seasons of low algal biomass or when it is of poor quality. Further, mothers should gain fitness benefits by producing fewer but higher quality offspring in harsh or competitive environments [4]. Because egg production increased but hatching success did not during the experiment, we assume that the feeding environment was optimal for the copepods, and that their condition even improved in the laboratory. It is possible that the egg quality in terms of maternal immunological or nutritional provisioning improved [7], [36], and that this explains the declining effect of pH difference on egg hatching. At the start of the experiment natural conditions were still influencing copepods. The change in pH conditions was possibly detrimental for these eggs. After the third day, the acclimatized copepods might have been able to produce higher quality and better performing eggs independent of the conditions they were facing. However, variation in hatching success was also higher on the last day. This may be caused by aging and other accumulating costs of living [15] and can complicate the detection of other effects.

In summary, our preliminary results demonstrate that temperature and pH scenarios predicted for 2100 may have significant effects on copepod reproductive output. Even though *Acartia* sp. copepods encounter changes in temperature and pH in their natural environment, the total egg production, as well as the total nauplii production, was lower at 20°C in low pH compared with ambient pH. Part of the negative effects may be combated with adaptive maternal effects, which seem to play a role in copepod environmental tolerance and reproduction. The ability of the mothers to invest in their offspring depends on their own condition, and the quality and quantity of their food. Thus the response of phytoplankton to climate change is highly important for copepods. As copepods constitute a major link between lower and higher trophic levels, their reproductive output can have ecological consequences on the whole ecosystem, including fish species of economical interest.
